# In vitro and in silico studies of silver nanoparticles (AgNPs) from *Allium sativum* against diabetes

**DOI:** 10.1038/s41598-022-24818-x

**Published:** 2022-12-21

**Authors:** D. Jini, S. Sharmila, A. Anitha, Mahalakshmi Pandian, R. M. H. Rajapaksha

**Affiliations:** 1 Department of Biotechnology, Malankara Catholic College, Mariagiri, Kanyakumari, Tamil Nadu India; 2 Department of Chemical Engineering, Hindustan Institute of Technology and Science, Chennai, Tamil Nadu India; 3 Center for Nanosciencesand Molecular Medicine, Amrita Vishwa Vidyapeetham, Kochi, Kerala India; 4 Department of Chemistry, Faculty of Science, University of Kelaniya, Kelaniya, Sri Lanka

**Keywords:** Biophysics, Biotechnology

## Abstract

In the present study, the silver nanoparticles (AgNPs) were synthesized from the bulbs of *Allium sativum*, characterized by UV–visible spectroscopy, FT-IR, SEM, HR-TEM, EDAX analysis and investigated its action on the inhibition of starch digestion. The results proved that the biosynthesized nanoparticles were uniformly dispersed, spherical shaped with the size ranging from 10 to 30 nm. The phytochemical and FT-IR analysis showed the presence of phenols, terpenoids, and amino acids in the synthesized AgNPs. The cytotoxicity analysis revealed that the synthesized AgNPs were non-toxic to the normal cells. The synthesized AgNPs exhibited significant free radical scavenging activity. The in vitro antidiabetic activity showed that the synthesized AgNPs increased glucose utilization, decreased hepatic glucose production, inhibited the activity of starch digestive enzymes such as α-amylase and α-glucosidase, and were not involved in the stimulation of pancreatic cells for the secretion of insulin. The in silico antidiabetic activity analysis (molecular docking) also revealed that the silver atoms of the AgNPs interacted with the amino acid residues of α-amylase, α-glucosidase, and insulin. The present study proved that the AgNPs synthesized from *A. sativum* have prominent antidiabetic activity in terms of reducing the hyperglycemia through the increased glucose utilization, decreased hepatic glucose production, and the inhibition of α-amylase and α-glucosidase enzymes. So it can be used as a promising nanomedicine for the treatment of diabetes.

## Introduction

Diabetes mellitus is a severe, prolonged, and complex metabolic disorder that results in hyperglycemia due to reduced insulin production (type I) or inefficient insulin utilization (type 2)^1^. Diabetes, the second largest disease, affected 25% of the global population. The number of diabetic patients is increasing day by day because of the change in lifestyle and various environmental factors^2^. In diabetic patients, the insulin will not be efficiently acted on the glucose metabolism which leads to the synthesis of a large amount of glucose in the liver by the action of glucagon^3^. Most of the synthetic medicines available for the treatment of diabetes have side effects and cannot be used during pregnancy^4^. The usage of medicinal plants for the treatment of diabetes begins from the time immortal. But they failed to cure the disease completely^5^. Although there are numerous medicines for the control and maintenance of normal blood sugar levels, there is no single medicine for the complete remedy of this disease. So the identification of an antidiabetic agent that has to stimulate insulin activity as well as to reduce/inhibit glucagon activity will be a major challenge for the modern scientific world.

The inhibition of dietary starch absorption is one of the remedial approaches to controlling hyperglycemia in type 2 diabetic patients. Alpha-amylase and alpha-glucosidase are the prime enzymes involved in the digestion and absorption of dietary starch^6^. Starch digestion can be suppressed by inhibiting those enzymes that lead to the delay of glucose uptake and therefore, decrease blood glucose levels in diabetic patients. Even though the drugs such as acarbose, and miglitol can inhibit the α-glucosidase and α-amylase activity, they produce undesirable side effects such as swelling, stomach uneasiness, diarrhea, and fart^7^. Therefore, the development of an effective and safe anti-diabetic agent is necessary for the treatment of diabetic patients.

The advent of nanotechnology leads to the synthesis of silver nanoparticles (AgNPs) from various medicinal plants. The phytocompounds (alkaloids, saponins, tannins, vitamins, phenolics, and terpenoids) and enzymes (hydrogenase, reductases) present in the plants serve as reducing and capping agents in the synthesis of silver nanoparticles^8^. The phytocompounds in the synthesized AgNPs are also responsible for their attractive and unique bioactive properties like antimicrobial, anticancer, and antidiabetic activities^9,10^ which make the nanoparticles to a wide range of applications in biomedicine^11^. Different medicinal plants with different therapeutic properties were used for the synthesis of AgNPs to increase their bioactive properties in a very low concentration^12,13^. The AgNPs synthesized from *Allium cepa* was used to treat numerous infectious diseases and cancer^14^. Several antidiabetic medicinal plants such as *Calophyllum tomentosum,* *Gymnema sylvestre*, and *Psoralea corylifolia* were used for the synthesis of AgNPs to increase their antidiabetic activities^2,15,16^.

*Allium sativum* (*A. sativum*), a traditional medicinal plant with a huge amount of medicinal properties^17^, showed better hypoglycemic activities in rats and humans^18^. The antidiabetic activity of *A. sativum* was well documented in terms of reducing insulin resistance and blood sugar level^19^. The sulphur-containing compounds (alliin, allicin, ajoene, diallyl disulfide, diallyl trisulfide, diallyl sulfide, S-allyl cysteine, and allyl mercaptan) from *A. Sativum* responsible for the pungent smell and have anti-diabetic activity^20^. The phytocompounds such as sitagliptin, vildagliptin, and alogliptin from *A. sativum* were used to treat diabetes clinically^21^.

The AgNPs were also synthesized from *A. sativum* which showed different bioactive properties like antimicrobial, anti-inflammatory, antioxidant, anticoagulant, anticancer and antidiabetic activities^22–25^. Even though there are reports in the synthesis of silver nanoparticles from *A. sativum*, there is no clear scientific evidence for the antidiabetic effect of AgNPs synthesized from *A. sativum*. So in the present study, the AgNPs were synthesized from *A. sativum,* characterized by UV–visible Spectrophotometer, FT-IR spectrophotometer, Scanning Electron Microscopy (SEM), EDAX (Energy Dispersive Spectroscopy), Transmission Electron Microscopy (TEM) and the anti-diabetic activity was evaluated by in vitro and in silico studies. It was proved that the synthesized AgNPs were reducing the hyperglycemia through the increased glucose utilization, decreased hepatic glucose production, and the inhibition of α-amylase and α-glucosidase enzymes.

## Results and Discussion

### Biosynthesis of silver nanoparticles

The AgNPs were synthesized from the bulbs of *A.sativum* by the chemical reduction of extract containing AgNO_3_ solution. According to the reports by Ahn et al.^26^, colour change is an important indicator in the synthesis of AgNPs. In this study also, during the synthesis of AgNPs, the colour of the reaction mixture was changed from yellow to brown which indicated the formation of silver nanoparticles. This colour change is due to the vibration of the Plasmon base, an optical property peculiar to noble metals^27^.

### Phytochemical screening

The preliminary qualitative phytochemical screening of the synthesized AgNPs and the *A. sativum* extract (Table 1) showed the presence of phytochemicals in both samples. The *A. sativum* extract displayed the presence of phytochemicals such as alkaloids, carbohydrates, phenol, protein, saponin, and terpenoids whereas the synthesized AgNPs showed the presence of phytochemicals such as phenol, protein, and terpenoids. The biomolecules present in the plants such as proteins, enzymes, alkaloids, phenolics, and tannins are responsible for the reduction and stabilization of silver nanoparticles. It was proved that an abundant amount of phytochemicals such as phenol, flavonoids, terpenoids, and proteins were present in the aqueous extract of garlic^28^. In the same way, the present study also revealed the presence of phytochemicals such as alkaloids, carbohydrates, phenol, protein, saponin, and terpenoids in the *A. sativum* extract. The results also showed (Table 1) that the phytochemicals such as phenol, protein, and terpenoids were transferred from the garlic extract to the AgNPs during the synthesis which may act as reducing and stabilizing agents during the synthesis. There are studies on the phytochemical screening of medicinal plants used for the AgNPs synthesis which reported that the phytomolecules served as both capping and stabilizing agents in the green synthesis of silver nanoparticles^29–32^. The AgNPs synthesized from the *Urtica dioica* leaves also showed the presence of phytochemicals such as proteins, phenols, diterpenes, and phytosterols^33^.

**Table 1:** Phytochemicals in the synthesized AgNPs and the *A. sativum* extract.

Phytoconstitutents	AgNPs	*A. sativum* extract
Alkaloids	** − **	** + **
Carbohydrate	** − **	** + **
Carboxylic Acid	**–**	**–**
Flavonoids	**–**	**–**
Phenol	** + **	** + **
Protein	** + **	** + **
Quinones	**–**	**–**
Saponins	** − **	** + **
Steroids	** − **	**–**
Tannins	**–**	**–**
Terpenoids	** + **	** + **

### Characterization of silver nanoparticles

#### UV–visible spectroscopy

**Figure 1:**
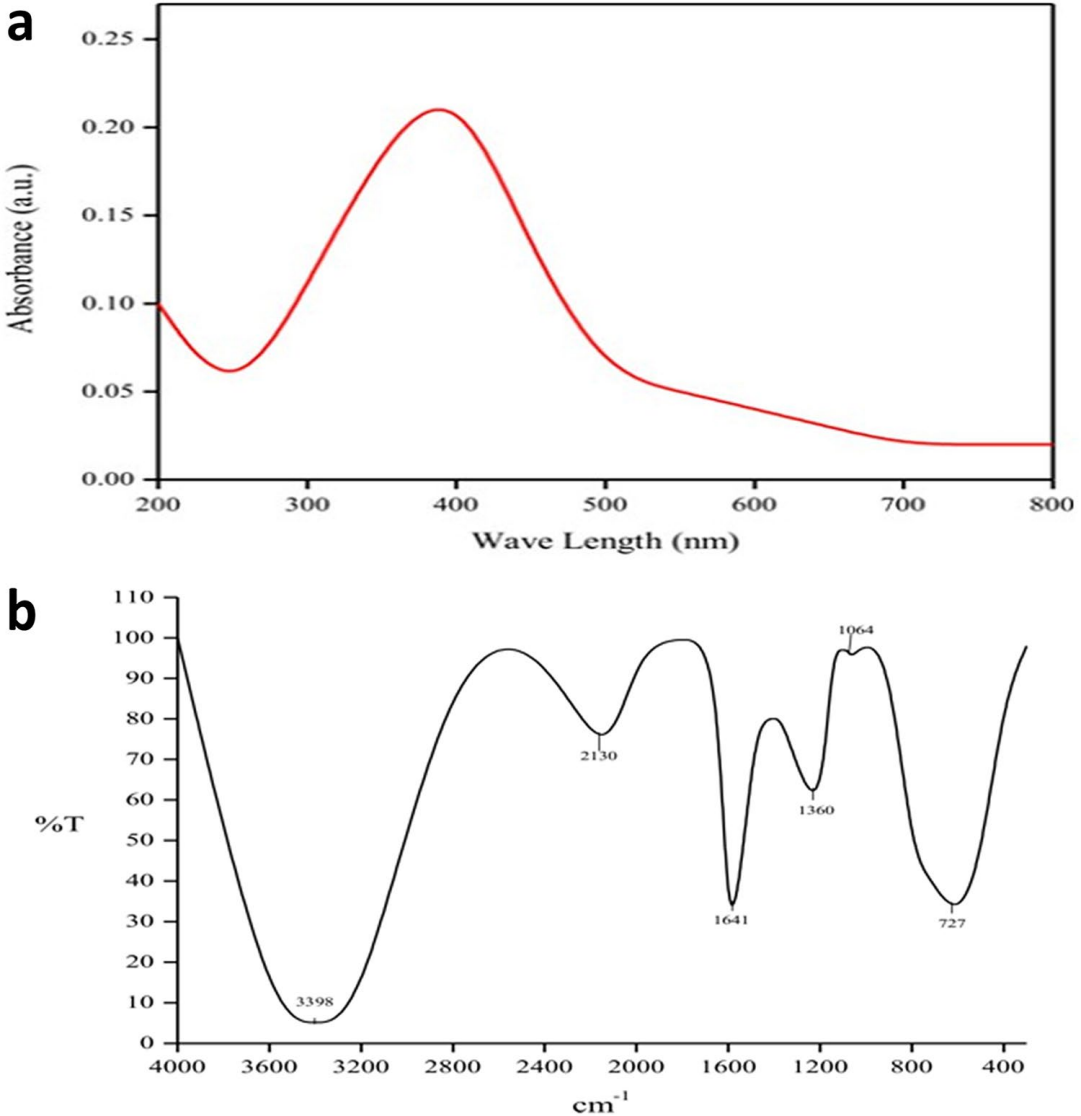
(**a**) Ultraviolet–visible spectrum of silver nanoparticles and (**b**) FT-IR spectrum of silver nanoparticles.

The synthesis of AgNPs was further confirmed by using UV–visible spectroscopy which was proved to be a vital technique for the characterization of nanoparticles. Generally, the peak of the UV–vis spectrum for the nanoparticles will be in the range of about 390 nm–470 nm which will be depended on the size, shape, and distribution of the nanoparticles^34^. The UV–vis spectrum of the synthesized AgNPs from *A. sativum* showed the major peaks at 400 nm (Fig. 1a) which was in the range between 390 and 470 nm confirmed the formation of nanoparticles in the reaction mixture. The formation of peaks at 400 nm was due to the localized surface plasmon resonance.

#### FT-IR spectroscopy

The presence of these phytochemicals in the synthesized AgNPs was further confirmed by the FT-IR spectrum data (Fig. 1b) which was recognized as an important tool in the characterization and determination of functional groups. The FTIR spectrum of the synthesized AgNPs showed the absorption peaks at 3398, 2130, 1641, 1360, 1064, and 727 cm^−1^ (Fig. 1b). The occurrence of N–H stretching (3398 cm^−1^) indicated the presence of primary amine groups of proteins. The presence of C≡C stretching (2130 cm^−1^), C = C stretching (1641 cm^−1^), O–H bending (1360 cm^−1^), C–O stretching (1064 cm^−1^), C = C bending (727 cm^−1^) revealed the existence of alkyne, alkene, phenol, ether, and alkane functional groups in the synthesized silver nanoparticles from *A. sativum* extract. The plants having alkaloids and polyphenolic compounds can reduce silver ions to silver nanoparticles and act as stabilizing agents. Recently there are numerous studies on the synthesis of AgNPs from the extracts of medicinal plants such as *Ribes khorassanicum*, *Cymbopogon citratus, Coptidis rhizome,* and *Bergenia ciliate* which showed the presence of phytochemicals in the synthesized AgNPs^35,36^.

#### Electron Microscopy

**Figure 2:**
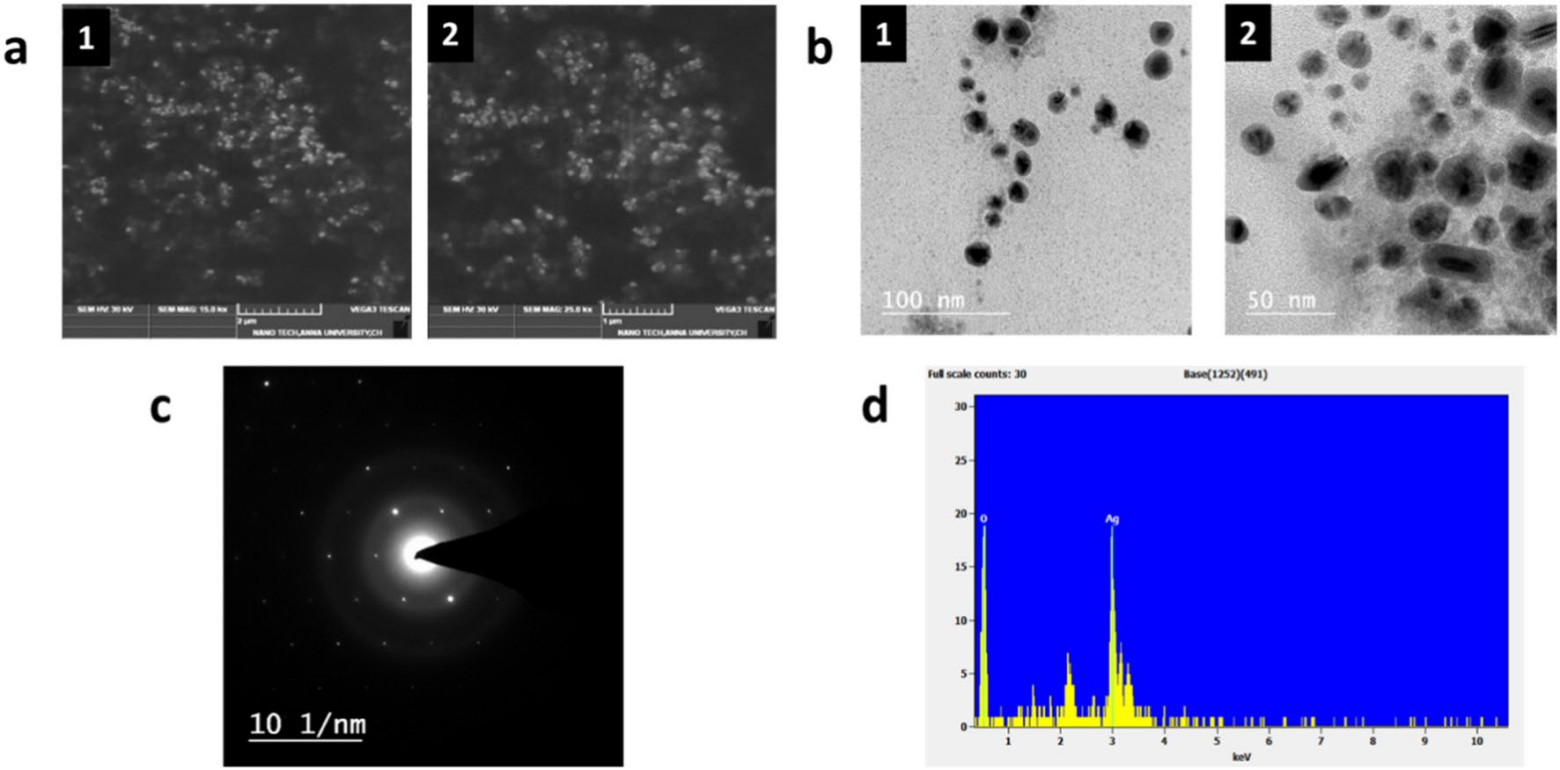
(**a**) SEM images of synthesized AgNPs (a1. 2 µm, a2.1 µm) (**b**) TEM images of synthesized AgNPs (b1. 100 nm, b2. 50 nm), (**c**) SAED pattern of synthesized AgNPs (**d**) EDAX spectrum of synthesized AgNPs.

The results of Scanning Electron Microscopy (SEM) and Transmission electron microscopy (TEM**)** analysis reconfirmed the presence of nanoparticles in the reaction mixture. Numerous studies mentioned that the AgNPs synthesized from medicinal plants showed a spherical shape and uniform distribution^37,38^. The results also exhibited that the synthesized nanoparticles were spherical in shape and were uniformly distributed (Fig. 2a, b). If the size of the synthesized silver particles is between 1 and 100 nm, it was considered nanoparticles^39^. The results also proved that the synthesized AgNPs have a size in the range of 30–100 nm.

The Selected Area Electron Diffraction (SAED) pattern showed that the synthesized AgNPs are highly crystalline (Fig. 2c). The sequence of spots in the selected area diffraction pattern will form the TEM image in which each spot represents a satisfied diffraction state of the nanoparticle’s crystal configuration. The different diffraction spots will be formed with respect to the different diffraction state that indicates the crystal nature of the specimen. The bright spot making a ring around the sample indicated the crystal nature of the sample.

#### Energy dispersive X-ray analysis

The elemental mapping by EDAX showed the presence of 93% silver and 7% oxides in the synthesized AgNPs. The EDAX spectrum showed a characteristic absorption peak at 3 keV (Fig. 2d) which may be due to the surface plasmon resonance of the metallic AgNPs^40^. A solid signal was obtained for silver along with a feeble oxygen peak in the EDAX profile (Fig. 2d). The oxygen peak may have started from the phytochemicals that are bound to the surface of AgNPs, showing the reduction of silver ions to AgNPs. The purity of the synthesized AgNPs was confirmed by the absence of other elements than silver (93%) and oxides (7%).

#### Cytotoxic analysis

**Figure 3:**
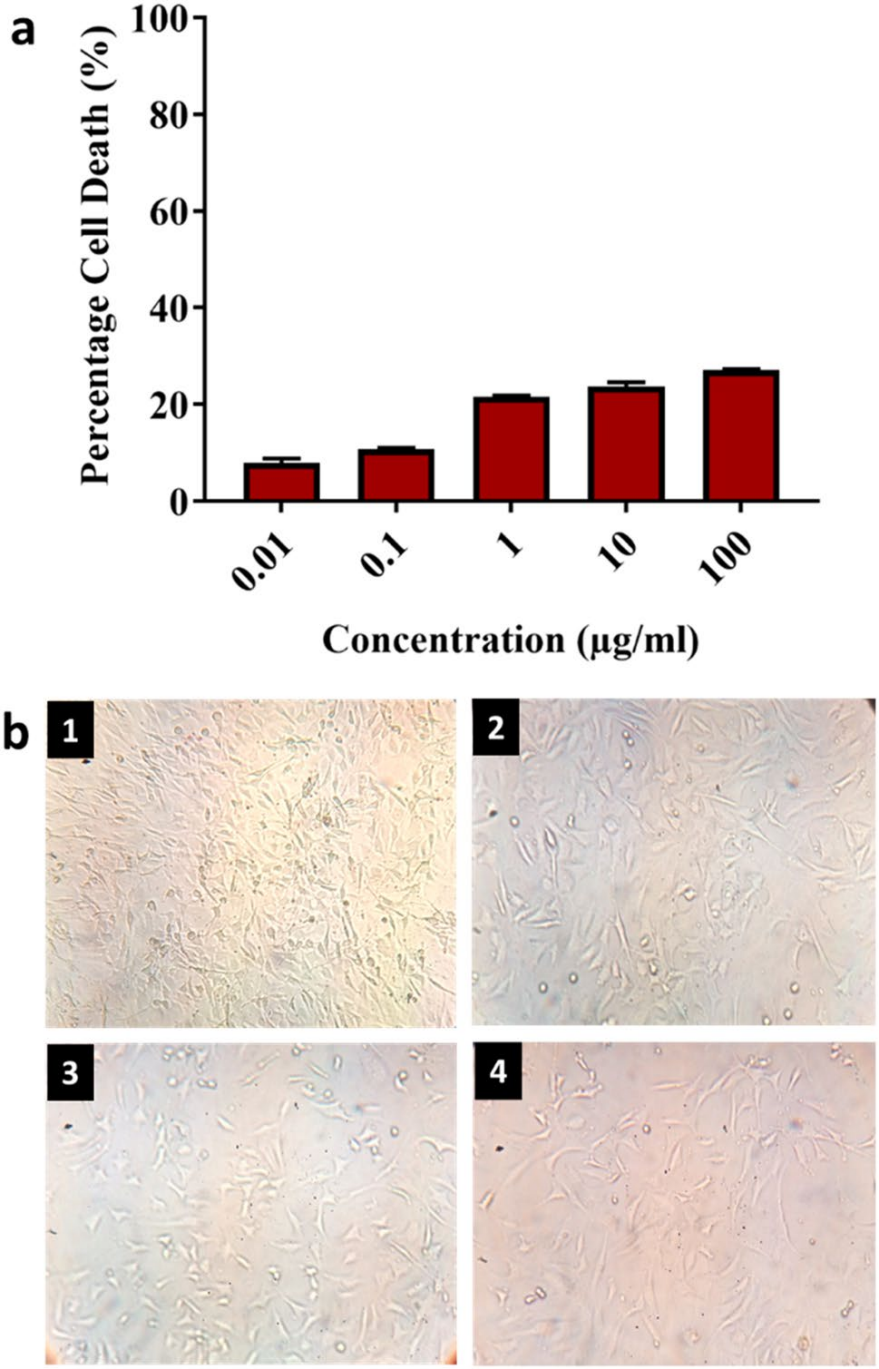
Cytotoxicity of the synthesized AgNPs from *A. sativum* extract. (**a**) Percentage of cell death, (**b**) Images of 3T3 Cell lines (b1. 3T3 untreated control cell lines, b2. 3T3 cell lines treated by 0.01 µg/ml AgNPs, b3. 3T3 cell lines treated by 1 µg/ml AgNPs b4. 3T3 cell lines treated by 100 µg/ml AgNPs).

The toxicity of the synthesized AgNPs at various concentrations was evaluated in 3T3 cell line using MTT (3-(4,5-dimethyl thiazol-2-yl)-2,5-diphenyl tetrazolium bromide) assay. The AgNPs were found to have less toxicity and the cell death was in a dose-dependent manner (Fig. 3a, b). The level of toxicity was increased along with the increased concentration of AgNPs. At the highest tested concentration of AgNPs (100 μg/ml), 27.26% cell death was observed which denoted that the synthesized AgNPs were non-toxic and can be used up to this concentration.

Testing the toxicity of the compound is the first step in the development of a drug for a particular disease. With regard to that, the cytotoxicity of the synthesized AgNP to normal 3T3 cell lines was evaluated by using a cell viability assay. The results proved that the synthesized AgNPs are non-toxic and the cell death was 27.26% at the concentration of 100 μg/ml (Fig. 3a, b). Several studies have shown the cytotoxicity of AgNPs in different cell lines^41^. The toxicity of the AgNPs depends on the size, shape, concentration, and nature of synthesis. Smaller size AgNPs (5 nm) were more toxic than the bigger size AgNPs (20-50 nm)^42^. The size of the AgNPs from *A. Sativum* is in the range of 10–30 nm which did not affect the normal 3T3 cells. The spherical-shaped AgNPs did not show any toxicity on the A549 cells^43^. In the same way, the AgNPs synthesized from *A.sativum* are also spherical in shape that may not cause any toxicity to the 3T3 cells. AgNPs synthesized from different plant sources are less toxic and show a higher level of bioactivity^44^ which denoted that the biosynthesized AgNPs are non-toxic in nature because of the presence of phytochemicals.

#### DPPH radical scavenging activity

**Figure 4:**
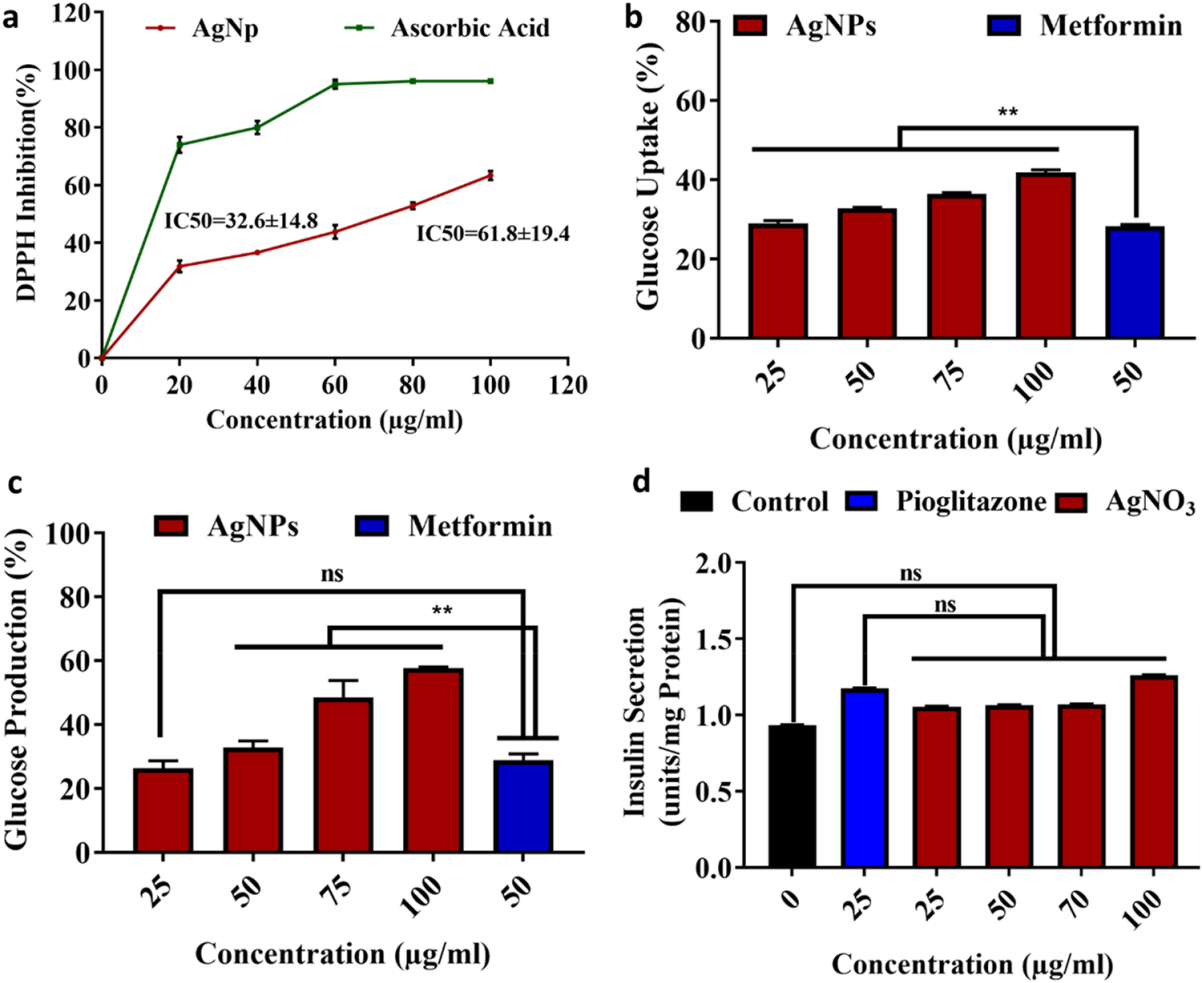
Activity of the AgNPs synthesized from *A.sativum* extract (**a**) Antioxidant activity (IC50: Concentration of the sample caused 50% DPPH radical scavenging ability), (**b**) Effect of AgNPs on glucose uptake in L-6 cell line, (**c**) Effect of AgNPs on glucose production in L-6 cell line and (**d**) Effect of AgNPs on Insulin secretion in pancreatic cells**. (**Data was represented as mean ± SD *P < 0.05; **P < 0.005; ns- nonsignificant).

The DPPH radical scavenging activity of AgNPs was displayed in Fig. 4a. The results confirmed that the synthesized AgNPs are free radical scavengers. As the concentration increased, the antioxidant activity increased in a dose-dependent manner. The AgNPs at concentrations ranging from 20 to 100 µg/ml exhibited the antioxidant activity of 31% to 63% with an average IC50 value of 61.81 ± 19.4. However, the AgNPs exhibited lower scavenging activity of DPPH than the standard ascorbic acid (IC50 value 32.63 ± 14.8).

Diabetes was connected with oxidative stress created by the synthesis of reactive oxygen species as a consequence of carbohydrate metabolism. The reactive oxygen species was playing a major role in the pathogenesis and development of diabetes^45^. So compounds with antioxidant activities will be more useful in the treatment of diabetes.DPPH is extensively used for testing the antioxidant activity of compounds or nanoparticles and offers easy and quick evaluation^46^. The antioxidant activity of the synthesized AgNPs was lesser than the standard ascorbic acid. The higher IC50 value (61.81 ± 19.4) of the synthesized AgNPs showed a lower scavenging activity than the ascorbic acid (32.6 ± 14.8) (Fig. 4a)^47^. The antioxidant activity of the biosynthesized AgNPs may be due to the presence of phytochemicals from *A. sativum* on the surface of the AgNPs (Table 1). The antioxidant activities of the phytochemicals help the cells to protect them from oxidative damage by free radicals^48^. Several studies reported that the AgNPs synthesized from different plant sources such as *Cleistanthuscollinus*^49^, *Elephantopuss caber*^50^, and *Terminalia species*^51^ increased the antioxidant activities.

### Antidiabetic activity by in vitro analysis

#### Glucose uptake assay

The utilization of Glucose by the L-6 cell lines was investigated in vitro. The results showed that the AgNPs increased the uptake of glucose over control at all the tested concentrations (Fig. 4b). As the concentration of AgNP increased from 25 to100 μg/ml, the glucose uptake level also increased from 28.9 to 41.5%. The glucose uptake ability of AgNPs was compared with metformin (standard diabetic drug) as reference. The results showed that the AgNPs displayed significantly higher glucose uptake ability (32.4%) than the metformin (28.8%) at 50 μg/ml concentration.

L6 cells are the model skeletal cells to study the mechanisms of glucose uptake by muscle cells. Skeletal muscle is the abundant tissue in the entire body and is responsible for the usage of postprandial glucose^52^. In addition, the uptake of glucose in the skeletal muscle was stimulated by the insulin and contractile activities and with the help of glucose transporter. The main pathological characteristics of non-insulin-dependent diabetes (type 2 diabetes) were the inhibition of this insulin stimulatory glucose uptake^53^. The present study proved that the synthesized AgNPs enhanced the glucose uptake ability of skeletal muscle cells over metformin (Fig. 4b). This might be due to the impact of AgNPs on the various receptors situated in the skeletal muscle cells^54^. The phytochemicals in the AgNPs may also interact with the skeletal muscle receptors for the increased uptake of glucose^55^.

#### Inhibition of hepatic glucose production

The effect of biosynthesized AgNPs on the inhibition of hepatic glucose production was determined in HepG2 hepatic cells after the treatment with glucagon. The results proved that the biosynthesized AgNPs significantly inhibited glucose production over the control at all the tested concentrations (Fig. 4c). The percentage of glucose production was inhibited from 26.28 ± 2.32 to 57.74 ± 0.77% at the AgNPs concentration of 25–100 μg/ml. It was also noted that the inhibition of glucose production by the AgNPs at 50 μg/ml was higher (32.78 ± 2.11%) than the standard diabetic drug metformin (26.89 ± 2.02%).

The hepatic liver cells are having a major role in retaining glucose homeostasis. Glucagon is playing a major role in the production of glucose from the stored form of carbohydrate, glycogen by glycogenolysis^56^. In type 2 diabetic patients, glucagon will be acting as a prime source for the production of glucose. At the same time, insulin will be insensitive (inefficient) in type 2 diabetic patients leading to hyperglycemia. The results proved that the AgNPs act on the glucagon to reduce its activity or inhibit hepatic glucose production.

#### Determination of insulin secretion in pancreatic cells

MIN6 pancreatic cells were used to check the effect of biosynthesized AgNPs on insulin secretion. The obtained results (Fig. 4d) showed that the AgNPs were not stimulated the MIN6 cells over the control to secret the insulin. The reference compound Pioglitazone at the concentration of 25 μg/ml secreted 1.18 units/mg of insulin. The biosynthesized AgNPs contributed to the synthesis of 1.06 units/mg of insulin which was insignificantly different from the control and the reference compound. As the concentration of AgNPs decreased from 25 to 100 μg/ml the insulin secretion level insignificantly differed from the control which denoted that the biosynthesized AgNPs were not involved in the insulin secretion.

In the case of type 2 diabetes, the insulin was inefficient or its quantity was less^57^. Since insulin is the prime hormone in the metabolism of glucose, its quantity was important in diabetic patients^58^. So the effects of AgNPs on the secretion of insulin by MNP6 cells were investigated. But the results showed that the biosynthesized AgNPs were not stimulated the MIN6 cells to secrete a large amount of insulin (Fig. 4d). Previous results proved that the synthesized AgNPs interact with the insulin to stimulate glucose uptake (Fig. 4b). At the same time, it also interacted with glucagon to inhibit the production of glucose. So the glucose uptake level was increased and the glucose production was decreased by the action of AgNPs. The outcome of this study clearly showed that the biosynthesized AgNPs triggered the glucose metabolism through its interaction with insulin or glucagon but could not act on the pancreatic cells to increase their insulin productivity.

#### α-amylase and α-glucosidase activities

**Figure 5:**
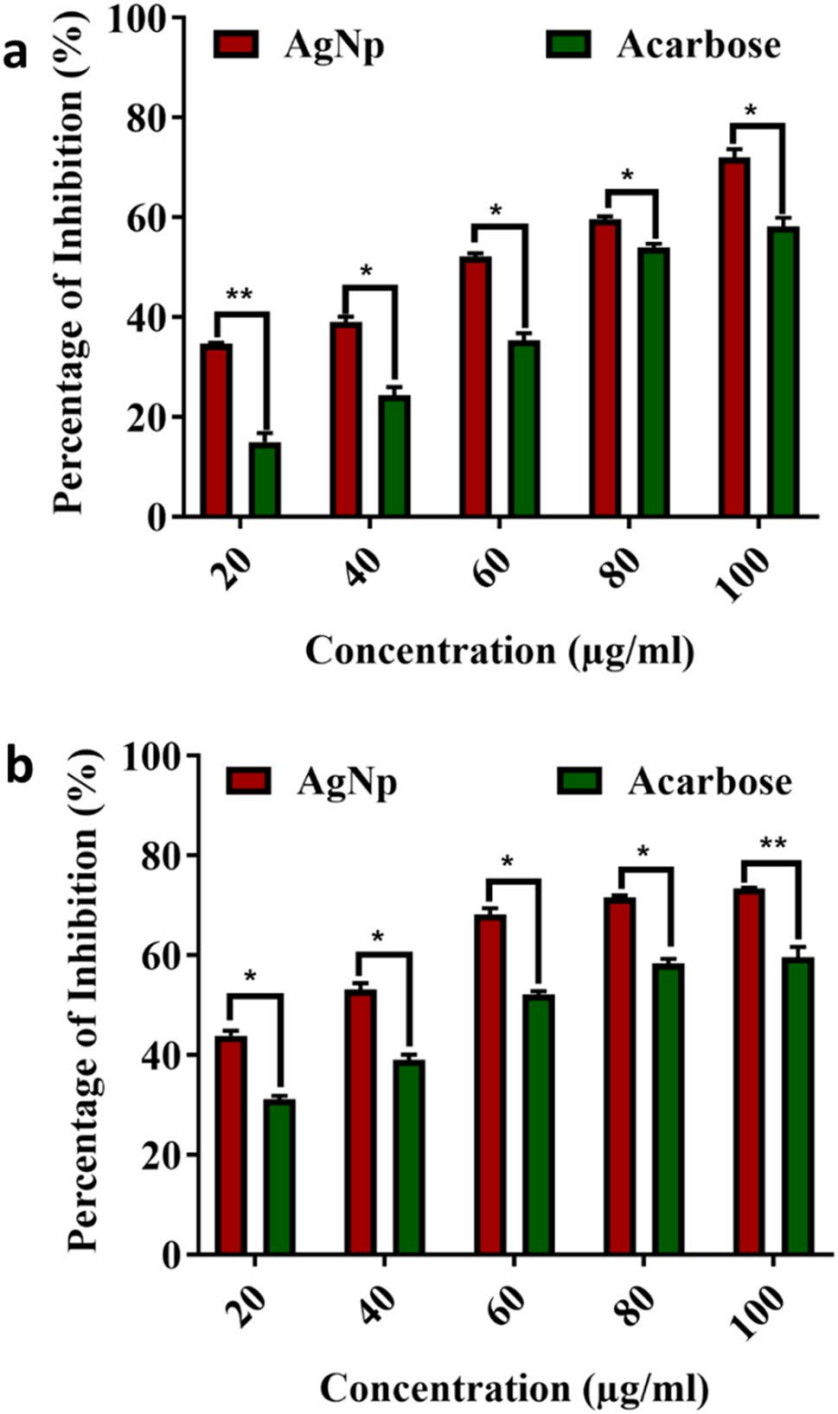
Inhibition of carbohydrate hydrolyzing enzymes by the AgNPs synthesized from *A. sativum* extract. (**a**) Inhibition of α-Amylase (**b**) Inhibition of α-Glucosidase. (Data was represented as mean ± SD *P < 0.05); **P < 0.005).

The inhibitory actions of AgNPs on α-amylase and α-glucosidase are shown in Fig. 5. The results showed that the biosynthesized AgNPs were inhibited significantly over the reference compound acarbose. The inhibitory activities of AgNPs were increased in a dose-dependent manner and it was significantly (*P* < 0.05) higher than the control.

In general, carbohydrates are the main ingredients of the human diet. α-amylase and α-glucosidase are the prime carbohydrate hydrolyzing enzymes that lead to the synthesis of a huge amount of glucose in the bloodstream that suppressed the uptake of glucose. So the inhibition of α-amylase and α-glucosidase leads to the reduction of blood glucose levels in diabetic patients. The results of this study proved that the biosynthesized AgNPs acted on both the enzymes and reduced the glucose level (Fig. 5a, b). It was already proved that the *A. sativum* is rich in phenolic compounds that acted as stabilizing agents during the synthesis of AgNPs. The reduction of enzyme activity may be due to the interaction of silver ions/ phytochemicals present in the biosynthesized AgNPs with the amino acids of carbohydrate hydrolyzing enzymes. This interaction was further confirmed by molecular docking and interaction analysis.

### Antidiabetic activity by in silico analysis

#### Molecular docking analysis

**Figure 6:**
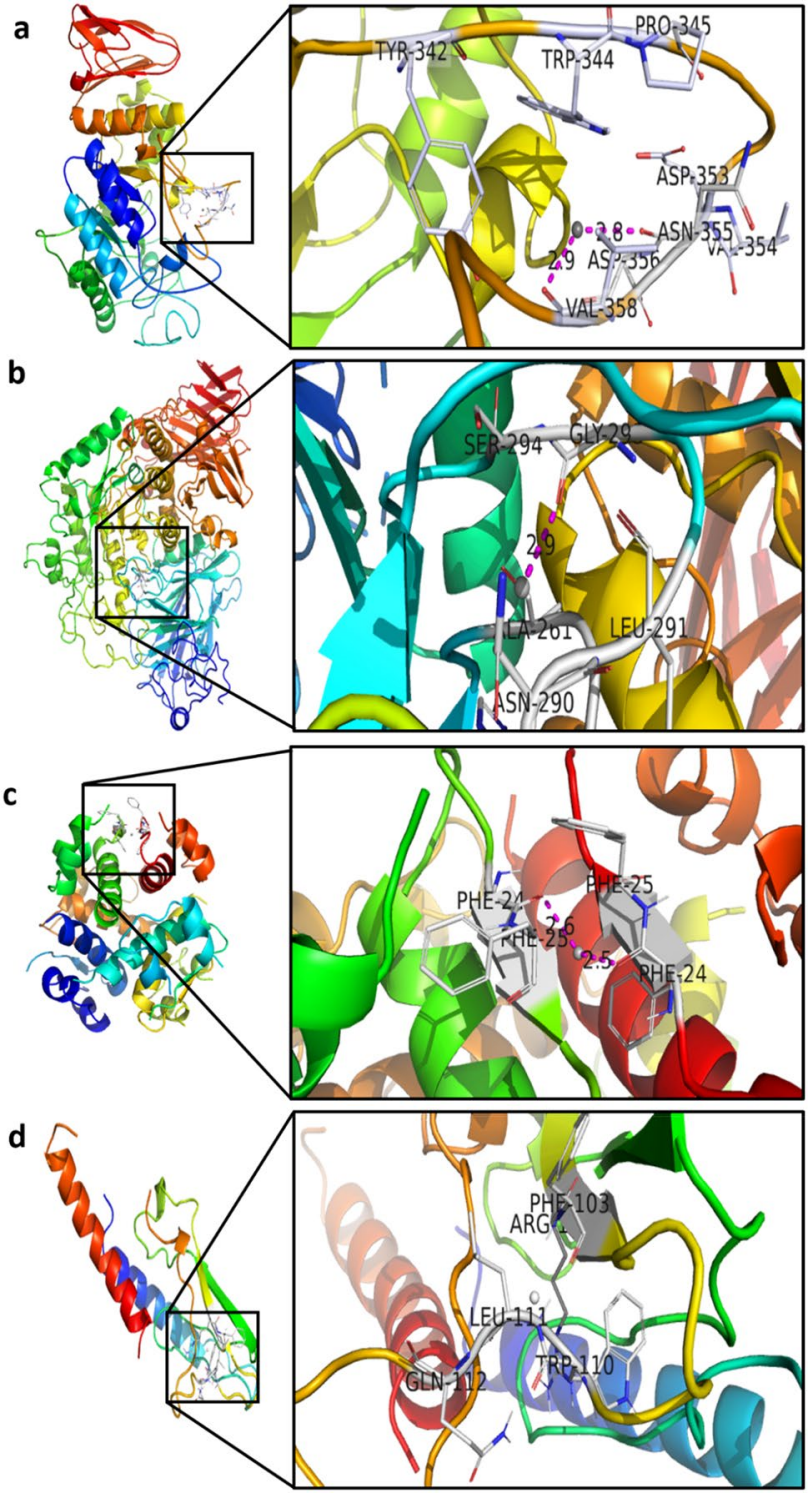
Molecular docking and Interactions between silver atoms and the amino acid residues of (**a**) α-Amylase (**b**) α- Glycosidase (**c**) Insulin (**d**) Glucagon (The binding of silver molecule with protein was shown in left side and the interaction between the amino acids residues and silver atom was shown in right side).

The molecular docking was done with the help of Autodock 4.2 to determine the biological interaction between the silver atoms and the protein targets such as α-amylase, α-glucosidase, insulin, and glucagon. The binding of silver atoms with the α-amylase, α-glucosidase, insulin, and glucagon during the docking procedure was given in Fig. 6. During the docking, the silver atom showed the binding energy of < 0 kcal/mol which revealed its free energy favouration to interact with the binding residues of the target proteins (Table 2). During the docking procedure, 100 docking poses were generated and all of those 100 instances were pointed to one binding site in each protein indicating the high affinity of silver neutral atom/s to these sites. According to Hevener et al.^59^, the Root Mean Square Deviation (RMSD) value should be < 2.0 Å for a valid docking pose. The re-docking results showed that all the docking poses have the RMSD value of < 1.0 Å which indicated a valid docking pose. The interaction analysis showed that the constructed silver atoms are capable of interacting with the ASN355 and VAL358 residues of α-amylase, GLY293 residue of α-glucosidase, and PHE24F and PHE24L residues of insulin through metal chelation. But the silver atom did not show any interaction with the glucagon (Fig. 6, Table 2).

**Table 2:** Molecular docking analysis of silver molecules.

Macromolecule	Binding energy (cal/mol)	Inhibtion constant ki (mM)	Metal chelating residues	Distance (Å)
α amylase	** − **0.52	415.35	Ag—ASN355 Ag—VAL358	2.82 2.90
α glucosidase	− 0.49	436.94	Ag—GLY293	2.86
Insulin	− 0.52	415.35	Ag—PHE24F Ag—PHE24L	2.62 2.54
Glucagon	− 0.53	408.39	–	

The interaction of metal ions like silver with different biomolecular targets leads to the inhibition of various biological activities such as inhibition of antioxidant enzymes, anti-metastasis, and anti-biofilm activities^60^. Among the 20 amino acids, cysteine, aspartic acid, and tyrosine exhibited the highest binding energy values with the silver ions^61^. The results of this study showed that the silver atoms interact with the asparagines, valine amino acid residues of α-amylase, Glycine residue of α-glucosidase, phenyl alanine residue of insulin through metal chelation (Fig. 6, Table 2). The search algorithm analyses and generates ligand pose at a target’s binding sites, taking into consideration final intermolecular energy, Van der Waal interaction energy, Hydrogen bond energy, dissolve energy, electrostatic energy, roto-translational and internal degrees of freedom. As this is a single silver atom, torsional energy can be neglected. In contrast to organic ligands, the binding energies showed by a single silver atom may seem low, but it is important to note that even a single silver atom can show free energy favoration to bind with amino acid residues of carbohydrate hydrolyzing enzymes and insulin. These binding energies will be significantly improved in the case of multiple silver atoms in the form of AgNPs. Even though the silver atom exhibited the binding energy of -0.53 with the glucagon, it did not show any interaction with amino acids (Fig. 6, Table 2) which showed that the silver molecules are not triggering the activity of glucagon. From the molecular docking analysis, it was revealed that the silver molecules can effectively interact with the carbohydrate hydrolyzing enzymes and insulin to reduce their activity.

## Methods

### Cell culture and maintenance

The four cell lines (3T3 pre-adipocytes cells, L6 muscle cells, HepG2 Liver Hepatic Cells, and MIN6 Pancreatic β- cells) used in this study were procured from National Centre for Cell Sciences (NCCS), Pune, India, and maintained in Dulbecco’s modified Eagles medium (DMEM) (Sigma Aldrich, USA).

### Preparation of garlic extract

*A. sativum,* bulbs (garlic) were collected from the local vegetable market in Chennai, India. The use of the *A. sativum* bulb in the present study complies with our institutional guidelines. The collected garlic bulbs (100 g) were peeled, minced into small pieces, and immersed in deionized water (200 ml) for 16 h. The extract was filtered using and used for the synthesis of AgNPs.

### Biosynthesis of silver nanoparticles and its phytochemical screening

The garlic extract (25 ml) was added to 50 ml of 0.1 M aqueous silver nitrate (Sigma-Aldrich, USA) solution at room temperature and stirred on a magnetic plate for 10 min. The solution thus obtained was kept in dark for the reduction of silver nitrate to silver nanoparticles. The colour change from light yellow to dark brown was used as an indication of the phyto reduction. The synthesized AgNPs were collected by centrifugation at 2323rcf for 30 min (10 °C) and washing with methanol and distilled water. The synthesized AgNPs were kept for drying at 20 °C using hot air oven. The dry powder was used for further analysis.

The presence of phytochemicals (alkaloids, terpenoids, saponins, flavonoids, tannins, steroids, phenolics, carboxylic acid, quinines, carbohydrates, proteins) in the synthesized AgNPs and the *A. sativum* (garlic) extract were examined using the standard biochemical procedures^62^.

### Characterization of silver nanoparticles

The biosynthesis of the AgNPs was further confirmed by using Ultraviolet–visible spectroscopy (Techcomp. Ltd.). FT-IR spectrophotometer (4000–400 cm^-1^) was used to identify the presence of possible biomolecules in the synthesized silver nanoparticles. The morphology and size of the synthesized silver nanoparticles were determined by Scanning Electron Microscopy (SEM-vega 3 tescan) and the selected area electron diffraction (SAED) pattern was obtained by High-Resolution Transmission Electron Microscopy (HR-TEM). The elemental detection was done by Energy Dispersive X-Ray Analyzer (Coxem, Korea).

### Cytotoxicity of AgNPs

A cell viability test was used to check the cytotoxicity of the synthesized AgNPs^63^. The 3T3 cells were grown in 96-well plates at the density of 5 × 10^3^ cells/well in the presence of 200 µl of DMEM with 10% FBS. After getting particular cell density, silver nanoparticles (0.01 μg, 0.1 μg, 1 μg, 10 μg, 100 μg) were added along with fresh DMEM and incubated for 48 h. Then 10 µl of MTT (5 mg/mL) was added and the cells were incubated for 4 h at 37 °C. The MTT solution was then disposed of and incubated in dark for forty minutes after the addition of DMSO (100 µl). The absorbance was recorded at 570 nm using a Scanning multi-well Spectrophotometer.

### DPPH radical scavenging activity

The AgNPs at different concentrations (25 to 100 μg/mL) were mixed with 1 ml of 0.1 mM methanolic DPPH (2,2diphenyl 1 picrylhydrazyl) solution and incubated at 37 °C in dark condition for 30 min. The change in the absorption of the reaction mixture was measured by taking the optical density at 517 nm. The ability of AgNPs to scavenge DPPH radicals was determined by estimating the percentage of inhibition.

### Antidiabetic activity by in vitro analysis

#### Glucose uptake assay

The utilization of glucose by the L6 (Rat skeletal muscle) cells with the treatment of AgNPs was determined by glucose uptake assay^55^. The L6 cells were cultured in a 96-well plate (8X10^3^cells/well) and incubated for 48 h at 37 °C. After attaining 80% confluency, the cells were kept in DMEM without glucose for 24 h. Then, the AgNPs were added at different concentrations (25 to 100 μl/ml) and incubated for 24 h in DMEM containing 300 mM glucose. An untreated control with glucose (300 mM) was also maintained. The glucose uptake was estimated by using high sensitivity glucose oxidase kit (Coral Clinical Systems: Lot No; RGLU1091).

#### Hepatic glucose production

The effect of AgNPs on Hepatic glucose production was evaluated in HepG2 cells. HepG2 Liver Hepatic cells were cultured in a 24-well plate at 37 °C. After attaining 80% confluency, the cells were kept in glucose-free/serum-free DMEM media with different concentrations (25 to 100 μl/ml) of AgNPs for 30 min and incubated for 6 h after treating with 100 nM glucagon. An untreated control without glucose was also maintained. After incubation, cells were isolated and the glucose content was estimated by using a glucose kit (Erba Mannheim, Germany), and the absorbance was read at 505 nm (Agilent, USA).

#### Determination of insulin secretion in pancreatic cells

The ability of the synthesized AgNPs to enhance the secretion of insulin was checked in MIN-6 (Pancreatic β- cell line) cells with the reference, Pioglitazone. MIN-6 (Pancreatic β- cell line) cells were cultured in 25 cm^2^ tissue culture flasks with DMEM media supplemented with 10% FBS, L-glutamine, sodium bicarbonate (Merck, Germany), and the antibiotic solution containing: Penicillin (100 U/ml), Streptomycin (100 µg/ml), and Amphotericin B (2.5 µg/ml). Cultured cell lines were kept at 37 °C in a humidified 5% CO_2_ incubator (NBS Eppendorf, Germany). The cells were equilibrated for 2 h in 1X KRBH Buffer (119 mM NaCl, 4.74 mM KCl, 2.54 mM CaCl_2_, 1.19 mM MgSO_4_, 1.19 mM KH_2_PO_4_, 25 mM NaHCO_3_, 10 mM HEPES (pH 7.4), 0.1 g of Bovine Serum Albumin). After equilibration, cells were washed twice with 1X KRBH Buffer and stimulated for 1 h in 1X KRBH Buffer supplemented with 300 mM glucose in the presence/absence of different concentrations of AgNPs (25–100 µg/ml) along with the reference compound Pioglitazone. After the stimulation, the amount of insulin secreted was calculated using the indirect ELISA method.

#### α-amylase assay

DNS method was used to determine the α-amylase inhibition activity of the biosynthesized AgNP^57^. The sample (500 μl) at different concentrations (20 to 100 μg/ml) was incubated with 500 μl of α-amylase solution. After pre-incubation, 1% starch in 0.02 M sodium phosphate buffer (500 μl) was added and incubated for 10 min. The reaction was stopped by adding 0.5 ml of dinitro salicylic acid colour reagent and kept for 15 min in a boiling water bath. Then the reaction mixture was diluted by adding 5 ml of distilled water and the absorbance of the colour solution was measured at 540 nm. The blank was maintained without adding enzyme. Acarbose was used as a reference compound to evaluate the inhibitory activities of AgNPs.

#### α-glucosidase assay

The α-glucosidase inhibitory activity of the AgNPs was determined by estimating the 4-nitrophenol released from p-nitrophenyl α-D glucopyranoside^65^. The AgNPs (0.2 ml) at various concentrations (25 to 100 μg/ml) were added to the 1.0 ml of potassium phosphate buffer (0.1 M, pH: 6.8), followed by 0.2 ml of α-glucosidase enzyme. The reaction solution was pre-incubated for 5 min at 37 °C. Then 0.3 ml of 10 mM p-nitrophenyl α-D-glucopyranoside was added and incubated for 30 min. The addition of 100 mM sodium carbonate (2.0 ml) terminated the reaction. The α-glucosidase acted on p-nitrophenyl α-D-glucopyranoside to liberate p-nitrophenol which was estimated by determining the absorbance at 400 nm using a spectrophotometer.

### Antidiabetic activity by in silico analysis

#### Molecular docking analysis

The interaction of the synthesized AgNPs with the carbohydrate hydrolysing enzymes (α-amylase and α-glucosidase) as well as insulin and glucagon were studied by molecular docking analysis. The structures of these proteins were retrieved from the Protein Data Bank (PDB ID: 4GQR, 5NN5, 2OMI, and 5OTX respectively), which provides access to 3D atomic coordinates. The protein structures were prepared for docking by removing ligands and water followed by the addition of polar hydrogen and Kollman charges. The structure of a single silver atom was constructed and its energy minimization was done with the help of the General Atomic and Molecular Electronic Structure System (US). The docking was performed by autodock 4.2 software using 100 Genetic Algorithm runs, 300 population sizes, and 25 million of evaluations. In order to validate the docking results, re-docking was performed to calculate the RMSD/ super impossibility of docking poses. After docking, the interactions between the AgNPs and amino acid residues were analyzed at 3.5 Å cut-off with the Protein–Ligand interaction profiler (https://projects.biotec.tu-dresden.de/plip-web/plip/index).

### Statistical analysis

Graph Pad Prism software was used to analyze the data statistically. All the experimental data obtained from the biological and technical triplicates were represented as mean ± standard deviation. The One-way ANOVA with Tukey's post hoc comparison was used to determine the statistical significance (*P* < 0.05) of the data.

## Conclusion

The silver nanoparticles (AgNPs) have been successfully synthesized from the aqueous extract of *A. sativum* and were characterized using standard analytical procedures. The synthesized AgNPs were uniformly dispersed, spherical in shape, and crystalline in nature with the nanometer size range. The presence of phenols, terpenoids, and amino acids and their corresponding functional groups were detected in the synthesized AgNPs and were non-toxic up to 100 µg/ml. The biosynthesized AgNPs are the best free radical scavenger and showed higher levels of antioxidants and antidiabetic activity. Moreover, the synthesized AgNPs enhanced the glucose uptake ability of skeletal muscle cells (L6) and inhibited glucose production in HepG2 hepatic cells. Insulin and glucagon were playing a major role in the control of glucose levels in human beings. The irregulation of these hormones leads to hyperglycemia and diabetes. The results of this study proved that the synthesized AgNPs or their phytochemicals interacted with these hormones for the regulation of glucose metabolism. These results were further confirmed by molecular modelling and docking analysis which proved that the silver atoms in the biosynthesized AgNPs interacted with the amino acid residues of the α-amylase, α-glucosidase, and insulin for the regulation of glucose metabolism. So the synthesized AgNPs could be recommended as a good nanomedicine for the treatment of diabetes.

## Supplementary Information

Supplementary Information.

## Data Availability

All data generated or analyzed during this study are included in this published article (and its Supplementary Information files).
