# Supplementary material for: In vitro and in silico studies of silver nanoparticles (AgNPs) from Allium sativum against diabetes
Source: Sci Rep. 2022 Dec 21;12:22109. doi: 10.1038/s41598-022-24818-x (PMC9772310; doi:10.1038/s41598-022-24818-x)
Supplement: Supplementary file 1 — Supplementary Information. [file 41598_2022_24818_MOESM1_ESM.docx]

**SUPPLEMENTARY MATERIALS**

**Table 1: Cytotoxicity of the synthesised AgNPs from *Allium sativum* extract**.

| Concentration (μg/ml) | Cell Death (%) |
| --- | --- |
| 0 | 0 ± 0 |
| 0.01 | 7.888072 ± 0.976385 |
| 0.1 | 10.72065 ± 0.392806 |
| 1 | 21.5925 ± 0.261503 |
| 10 | 23.67391 ± 0.92456 |
| 100 | 27.10937 ± 0.146112 |

Data was represented as mean ± SD, n=3

**Table 2: Antioxidant activity of the synthesised AgNPs from *Allium sativum* extract**

| Concentration (µg/ml) | % of inhibition by AgNPs | % of inhibition by Ascorbic Acid |
| --- | --- | --- |
| 0 | 0 ± 0 | 0±0 |
| 20 | 31.86553 ± 2.075787 | 74.17929 ± 2.767716 |
| 40 | 36.60702 ± 0.745968 | 80.52482±2.230854 |
| 60 | 43.7799±2.3683 | 95.51768±1.51778 |
| 80 | 52.86998±1.164179 | 96.11493±0.532163 |
| 100 | 63.38467±1.561246 | 96.54938±0.744793 |

Data was represented as mean ± SD, n=3

**Table 3: Effect of AgNPs on glucose uptake in L-6 cell line**

| Sample | Concentration (µg/ml) | Glucose Uptake (%) |
| --- | --- | --- |
|  | 0 | 0 ± 0 |
| AgNPs | 25 | 28.97521 ± 0.740278 |
|  | 50 | 32.73621 ± 0.317892 |
|  | 75 | 36.42466 ± 0.354886 |
|  | 100 | 41.86735 ± 0.607135 |
| Metformin | 50 | 28.23581 ± 0.469382 |

Data was represented as mean ± SD, n=3

**Table 4: Effect of AgNPs on glucose production in L-6 cell line**

| Sample | Concentration (µg/ml) | Glucose production (%) |
| --- | --- | --- |
| AgNPs | 0 | 0 ± 0 |
|  | 25 | 26.27855 ± 2.329259 |
|  | 50 | 32.78072 ± 2.107858 |
|  | 75 | 48.57182 ± 5.255789 |
|  | 100 | 57.72432 ± 0.356931 |
| Metformin | 50 | 28.89093 ± 1.920134 |

Data was represented as mean ± SD, n=3

**Table 5: Effect of AgNPs on Insulin secretion in pancreatic cells**

| Sample | Concentration (µg/ml) | Insulin Secretion (units/mg of Protein) |
| --- | --- | --- |
| Control | 0 | 0.934157 ± 0.002817 |
| Pioglitazone | 25 | 1.175381 ± 0.001437 |
| AgNPs | 25 | 1.056467 ± 0.001989 |
|  | 50 | 1.064682 ± 0.002354 |
|  | 70 | 1.07183 ± 0.002781 |
|  | 100 | 1.260353 ± 0.003387 |

Data was represented as mean ± SD, n=3

**Table 6: Inhibition of α-Amylase by AgNPs**

| Concentration (µg/ml) | Percentage of Inhibition by AgNPs (%) | Percentage of Inhibition by Acarbose (%) |
| --- | --- | --- |
| 0 | 0 ± 0 | 0 ± 0 |
| 20 | 34.68063 ± 0.171221 | 14.99411 ± 1.743569 |
| 40 | 38.99055 ± 1.064886 | 24.2984 ± 1.678458 |
| 60 | 52.06065 ± 0.713013 | 35.32656 ± 1.422076 |
| 80 | 59.51279 ± 0.68046 | 54.00569 ± 0.706933 |
| 100 | 72.03404 ±1.563872 | 58.15841 ± 1.71515 |

Data was represented as mean ± SD, n=3

**Table 7: Inhibition of α-Glucosidase by AgNPs**

| Concentration (μg/ml) | Percentage of Inhibition by AgNPs (%) | Percentage of Inhibition by Acarbose (%) |
| --- | --- | --- |
| 0 | 0 ± 0 | 0 ± 0 |
| 20 | 43.86942 ± 0.95269 | 31.12518 ± 0.670479 |
| 40 | 53.09808 ± 1.279717 | 38.99055 ± 1.064886 |
| 60 | 68.18119 ± 1.205639 | 52.06065 ± 0.713012 |
| 80 | 71.5252 ± 0.482974 | 58.30623 ± 0.955483 |
| 100 | 73.43919 ± 0.097911 | 59.59389 ± 2.034447 |

Data was represented as mean ± SD, n=3


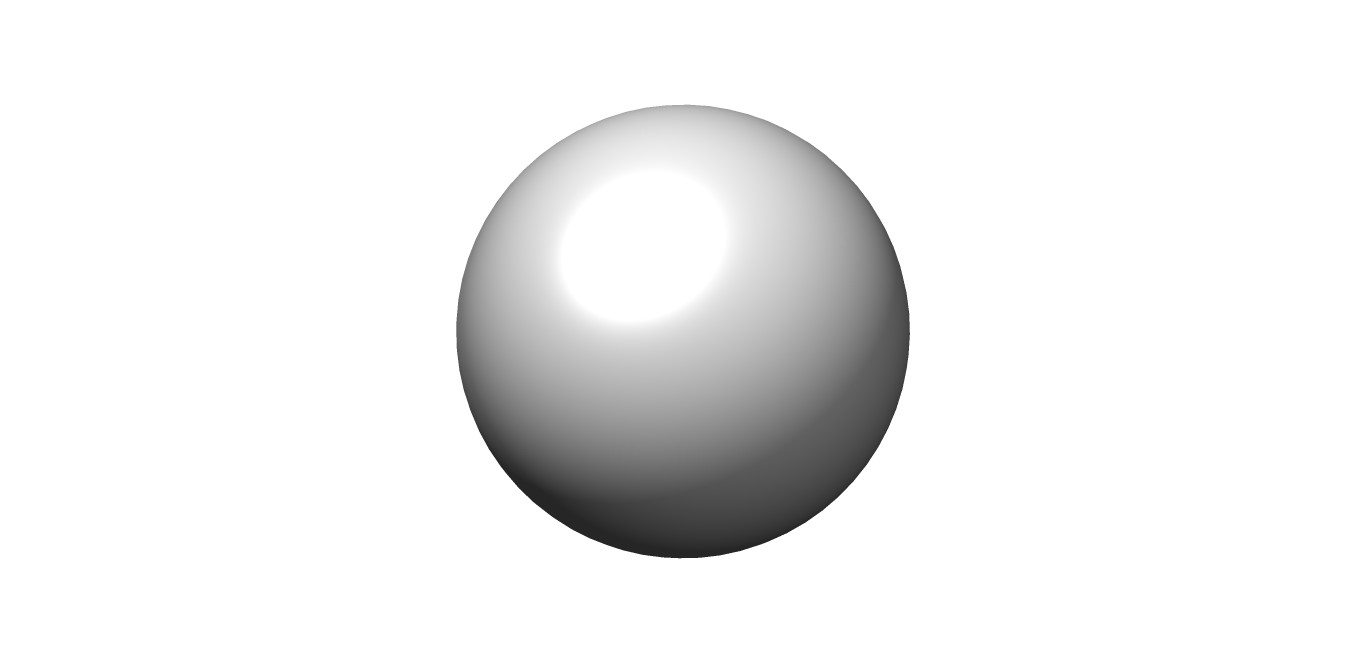


Figure 1: 3D-Image of the silver atom


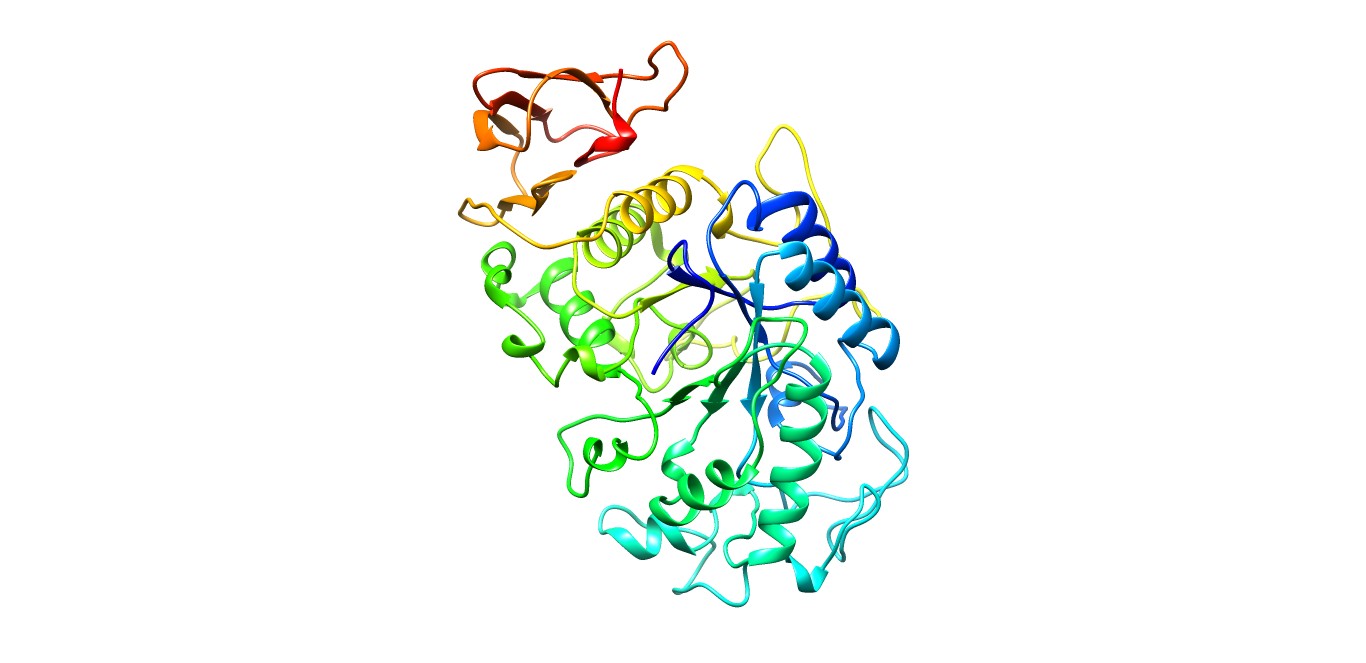


Figure 2: 3D image of α-Amylase


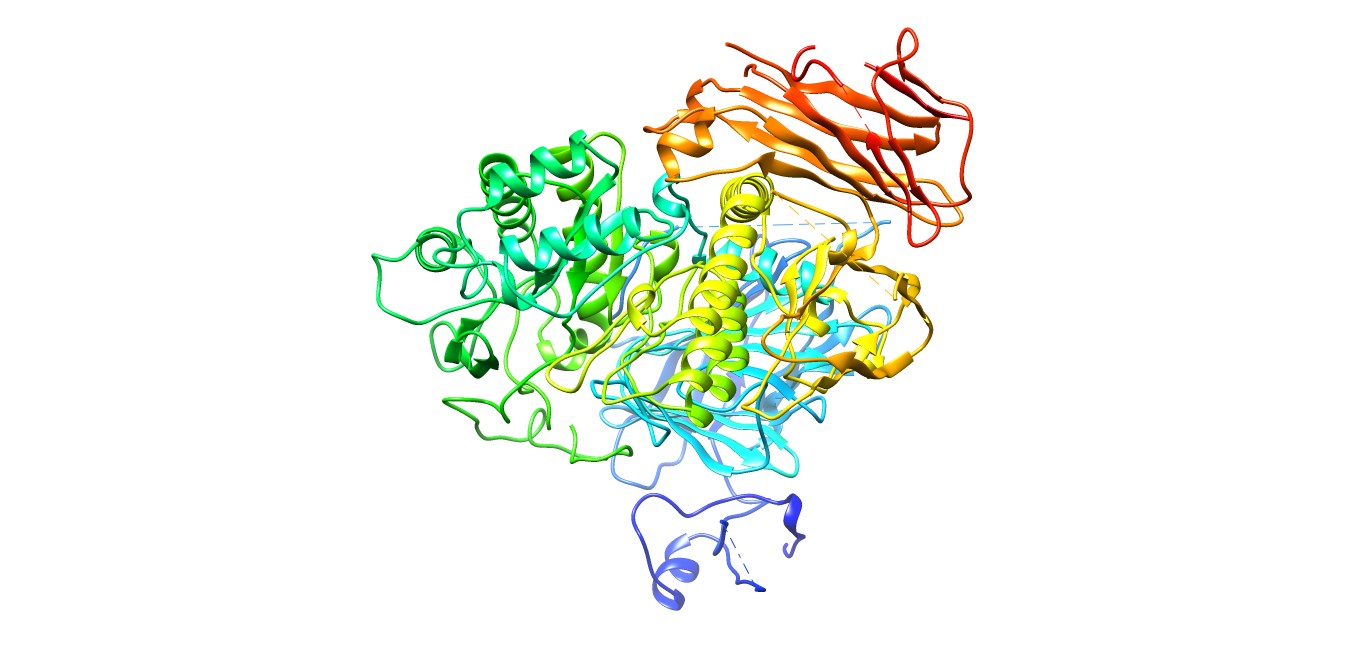


Figure 3: 3D image of α- Glycosidase


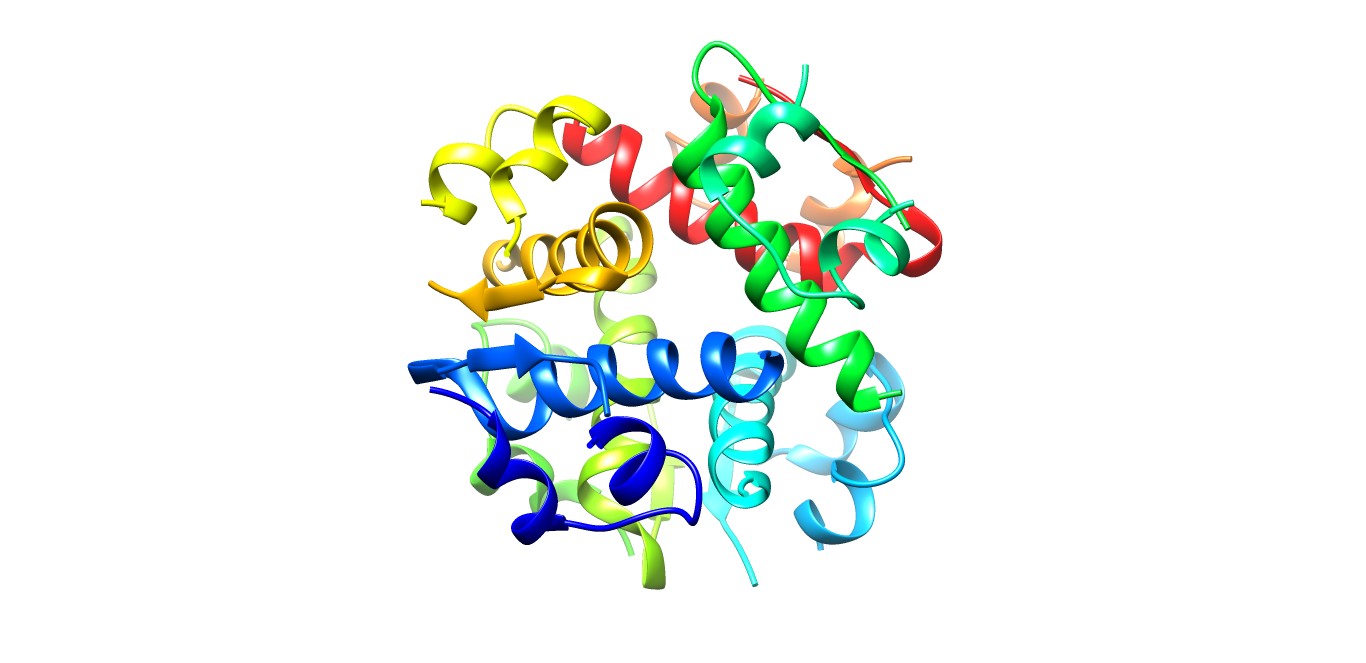


Figure 4: 3D image of Insulin


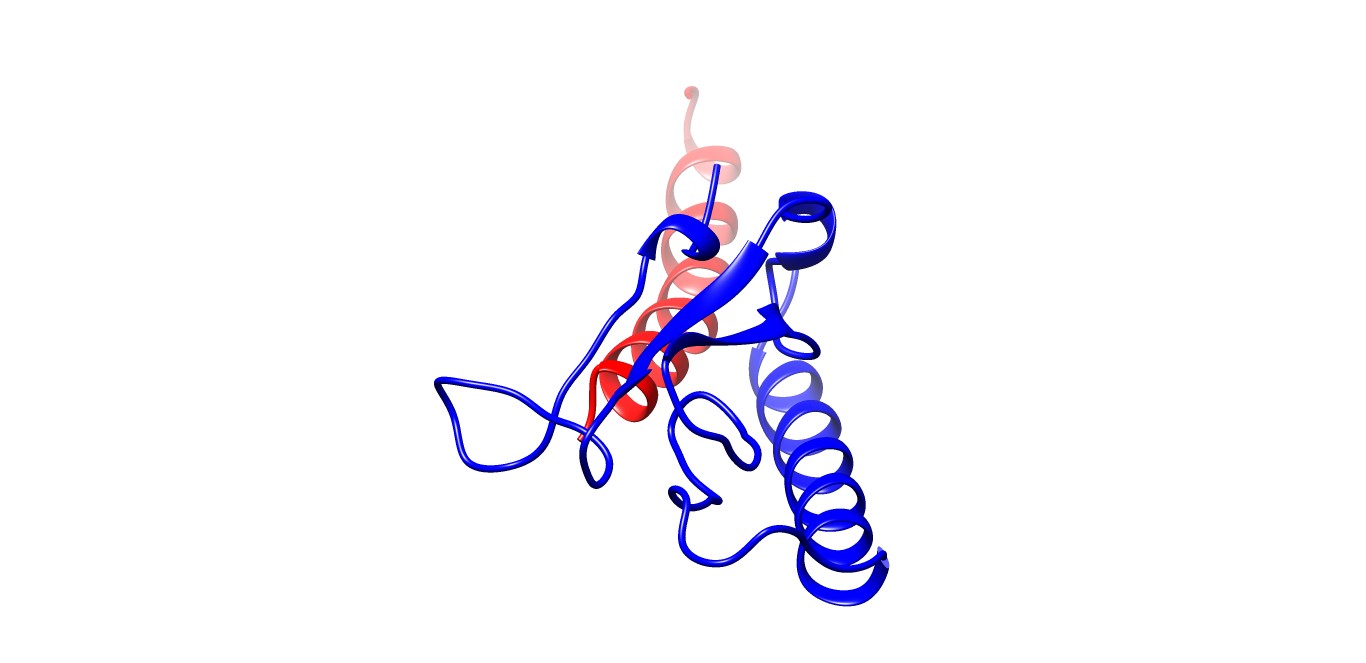


Figure 5: 3D image of Glucagon
